# Supplementary material for: Genetic Association between the Levels of Plasma Lipids and the Risk of Aortic Aneurysm and Aortic Dissection: A Two-Sample Mendelian Randomization Study
Source: J Clin Med. 2023 Mar 2;12(5):1991. doi: 10.3390/jcm12051991 (PMC10004011; doi:10.3390/jcm12051991)
Supplement: Supplementary file 1 [file jcm-12-01991-s001.zip › jcm-2214300-supplementary.pdf]

Journal of
Clinical Medicine

Article

Genetic Association between the Levels of Plasma Lipids and the Risk of Aortic Aneurysm and Aortic Dissection: A Two-Sample Mendelian Randomization Study

Rui Li[1,†], Chao Zhang[1,†], Xinling Du[1,*] and Shi Chen[2,*]

1 Department of Cardiovascular Surgery, Union Hospital, Tongji Medical College, Huazhong University of Science and Technology, Wuhan, China
2 National Clinical Research Center for Cardiovascular Diseases, State Key Laboratory of Cardiovascular Disease, Fuwai Hospital, National Center for Cardiovascular Diseases, Chinese Academy of Medical Sciences and Peking Union Medical College, Beijing, China
* Correspondence: xinlingdu@hust.edu.cn (X.D.); chenshi@fuwai.com (S.C.)
† These authors contributed equally to this work.

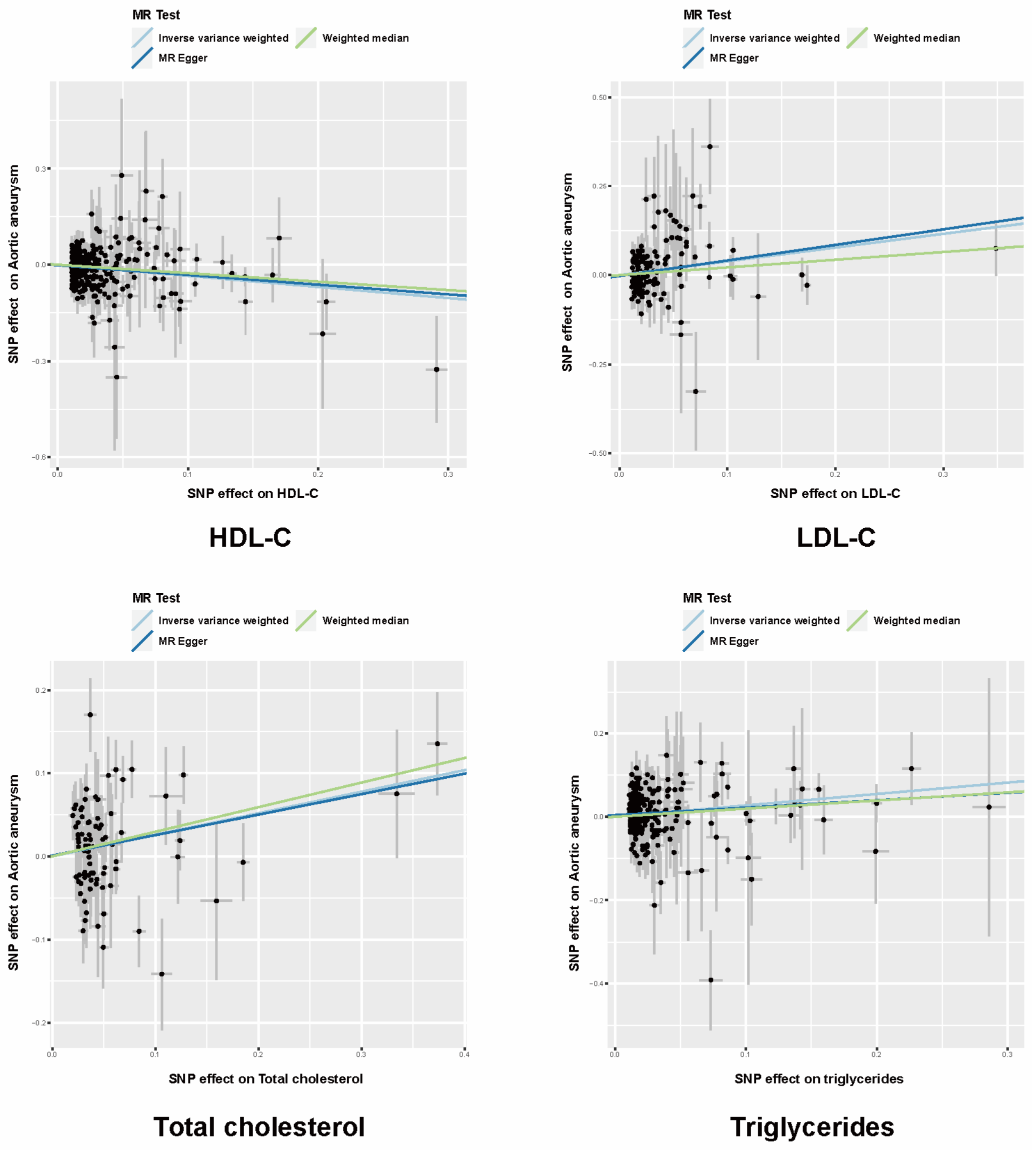

Figure S1. Scatter plots of the association between plasma lipids and the risk of aortic aneurysm.

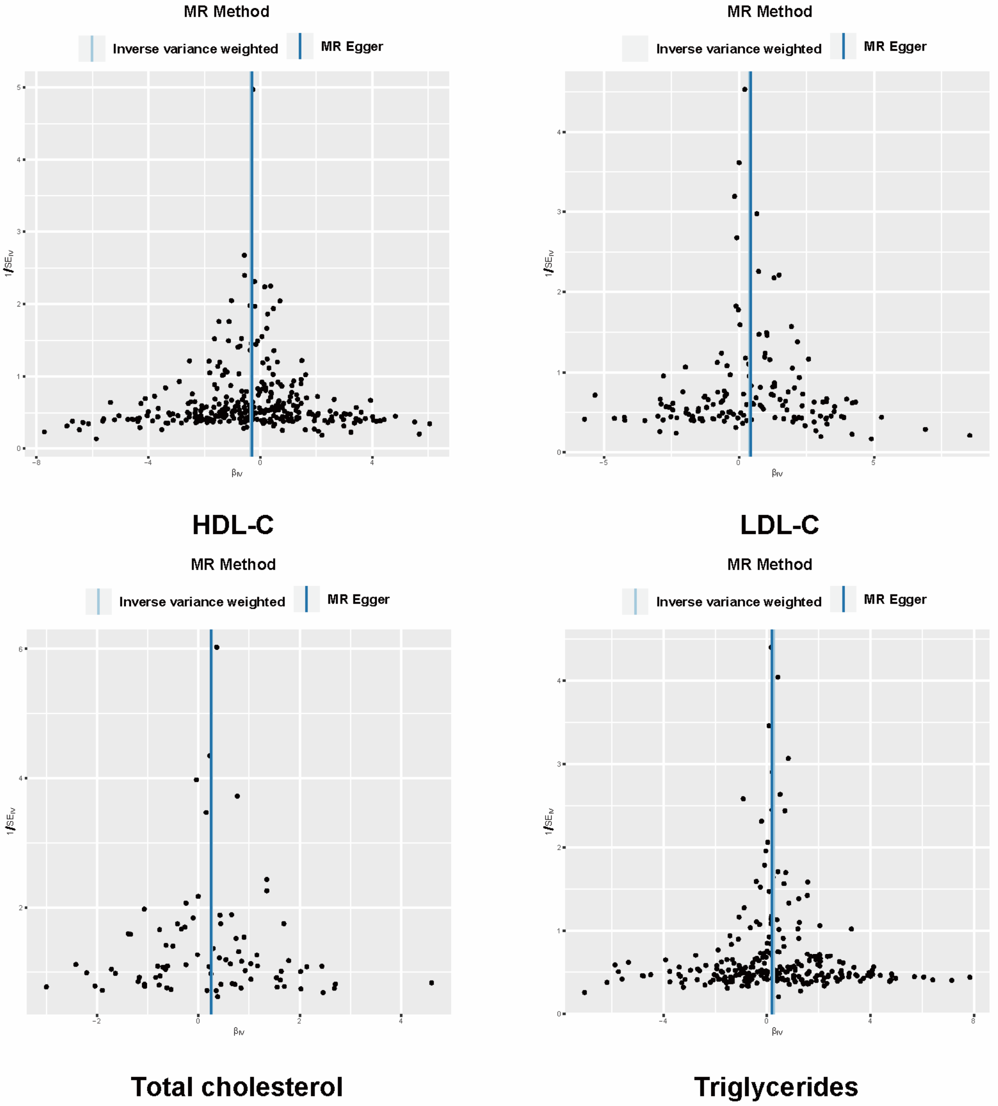

Figure S2. Funnel plots for the association of plasma lipids and the risk of aortic aneurysm.

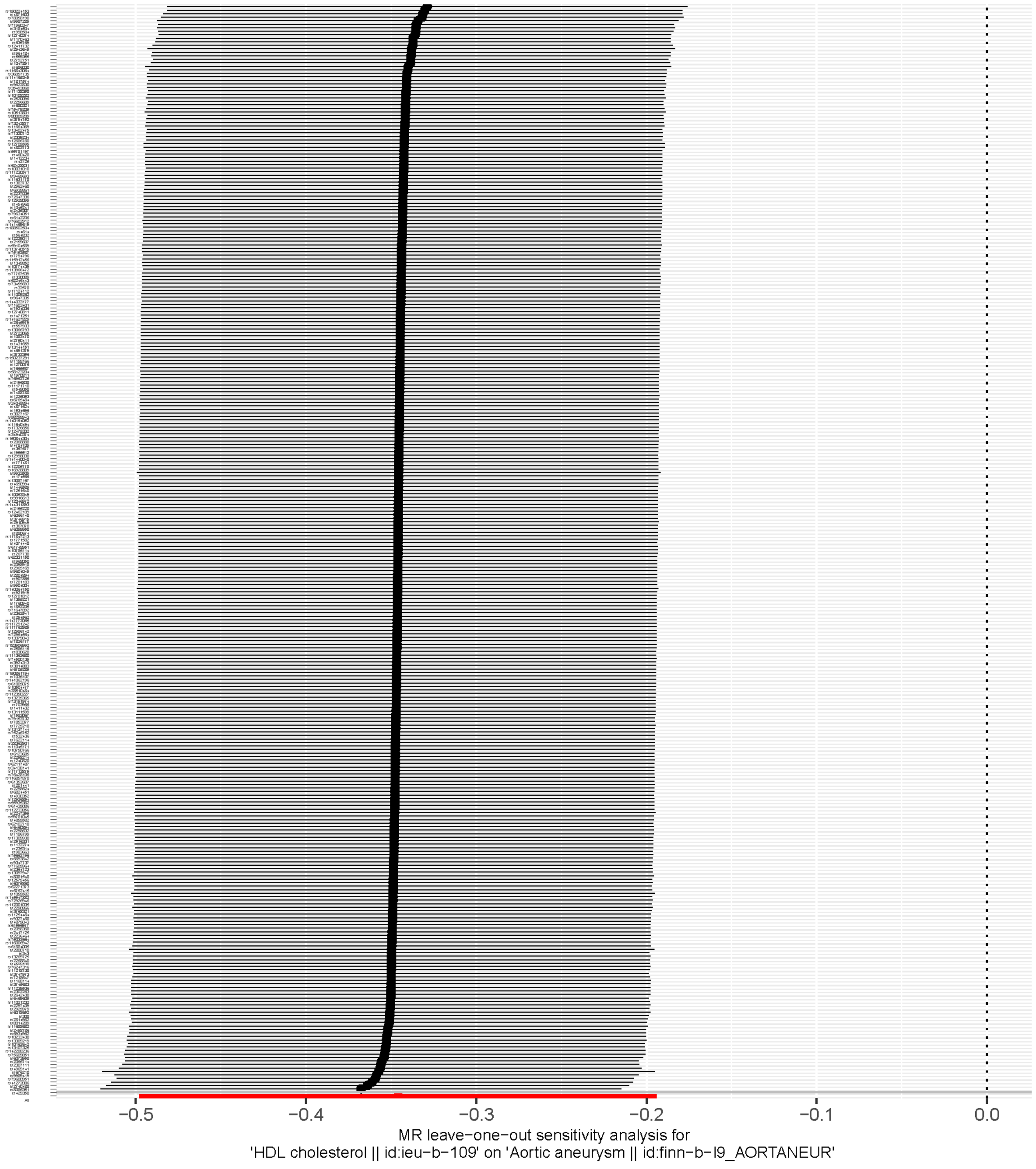

Figure S3. Leave-one-out analysis of the association between HDL-C and aortic aneurysm.

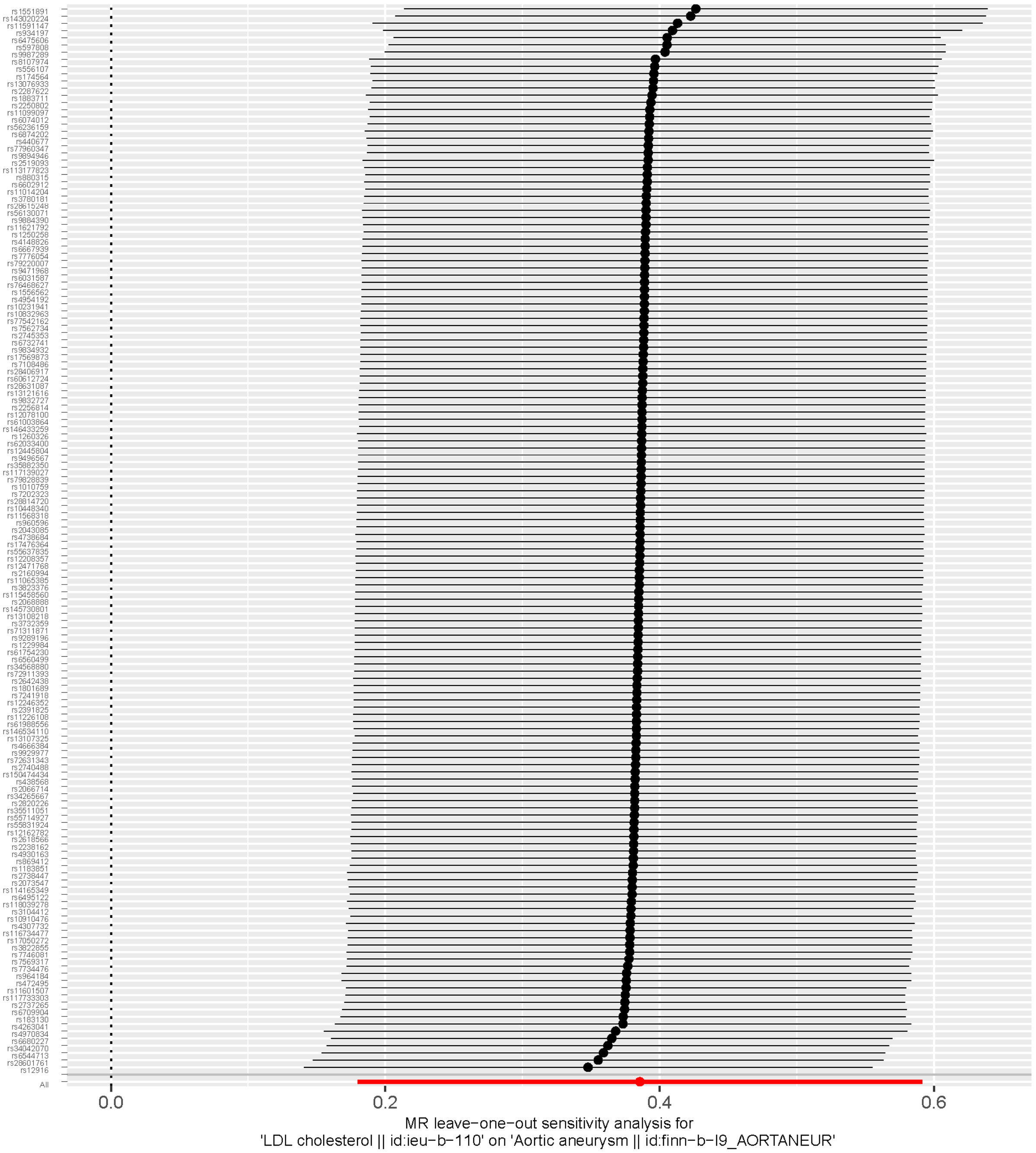

Figure S4. Leave-one-out analysis of the association between LDL-C and aortic aneurysm.

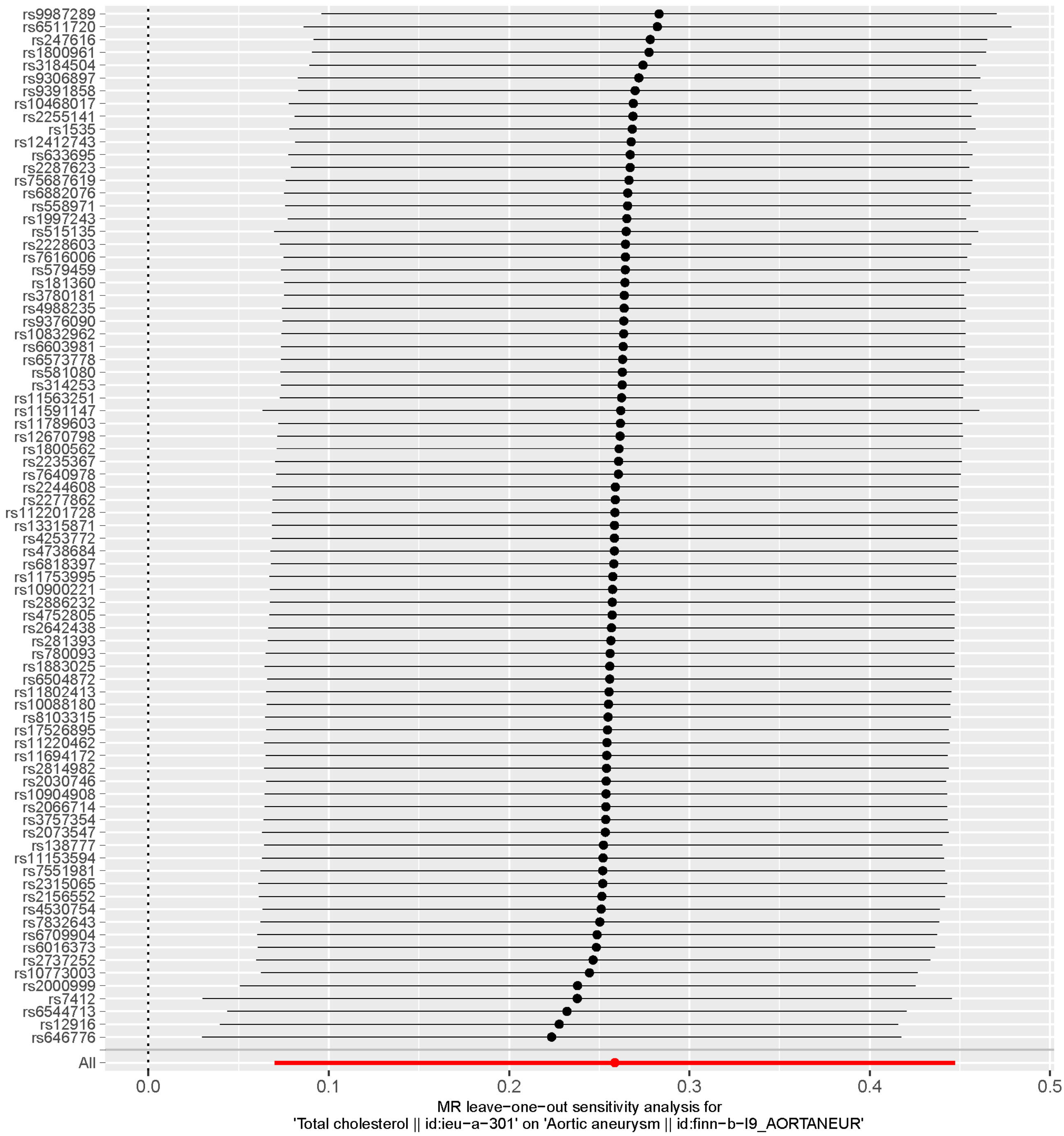

Figure S5. Leave-one-out analysis of the association between total cholesterol and aortic aneurysm.

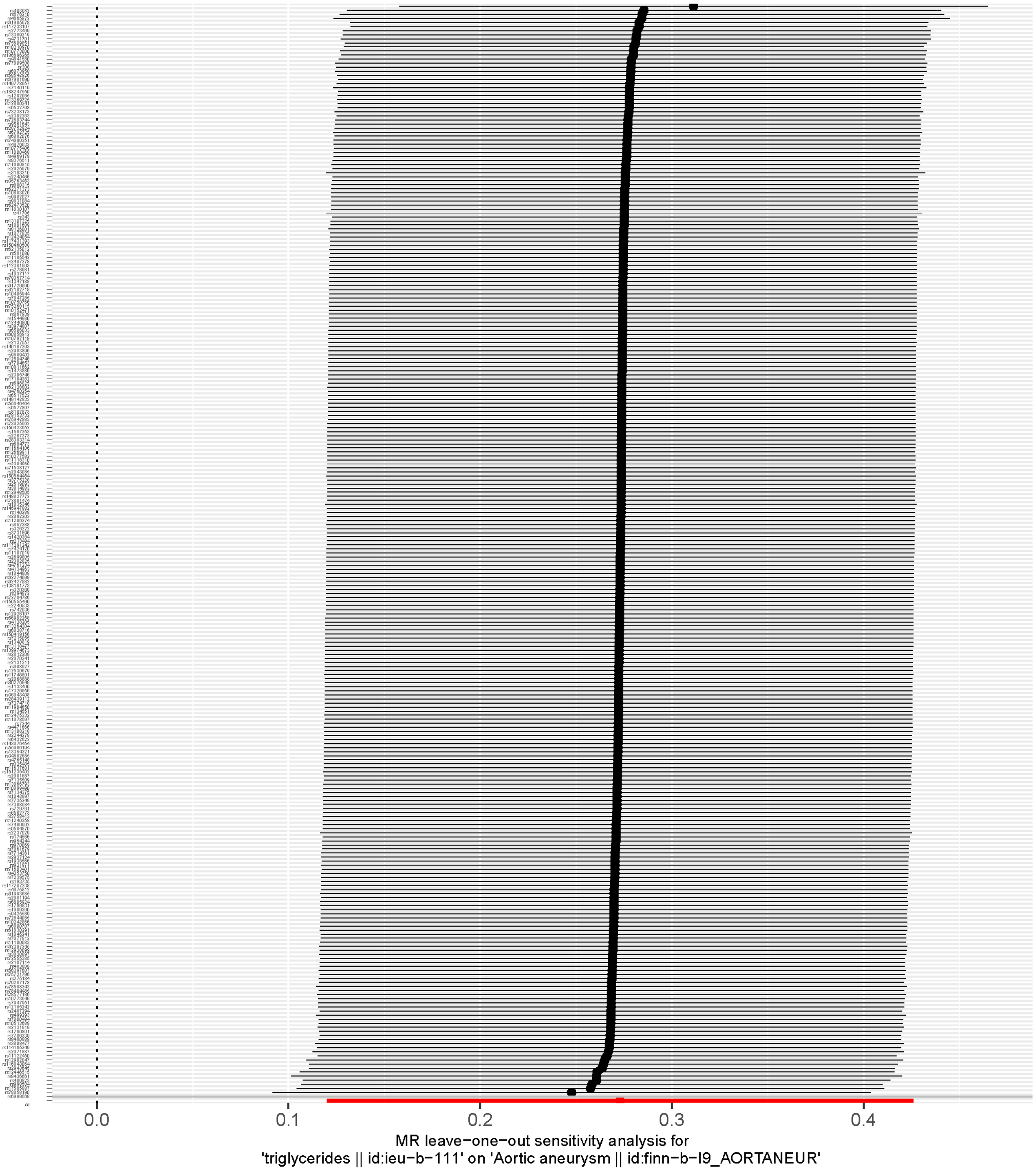

Figure S6. Leave-one-out analysis of the association between triglycerides and aortic aneurysm.

Table S1. SNPs of HDL-C extracted from UK Biobank with statistically significant threshold

rsID	A1	A2	BETA	SE	EAF	*p* value
rs114165349	C	G	-0.0810512	0.00636562	0.023124	3.90E-37
rs3768321	T	G	-0.0452296	0.00239482	0.196691	1.50E-79
rs12045101	T	C	-0.0145519	0.00222909	0.240857	6.70E-11
rs2642438	G	A	0.0276562	0.00207854	0.702698	2.10E-40
rs76962725	A	G	-0.0277897	0.00508558	0.037253	4.60E-08
rs185073199	A	T	0.0637585	0.0100718	0.010267	2.40E-10
rs75246752	C	G	0.0491144	0.00841625	0.012897	5.40E-09
rs1771582	G	T	0.0129458	0.00197832	0.557402	6.00E-11
rs4650994	A	G	-0.018056	0.00190061	0.531842	2.10E-21
rs61805075	A	G	-0.0257183	0.00202534	0.328694	6.00E-37
rs141440048	T	C	0.0436872	0.00756751	0.017665	7.80E-09
rs2298632	T	C	0.0144252	0.00193744	0.498073	9.70E-14
rs771481	A	T	0.0288148	0.0024568	0.18382	9.10E-32
rs3747973	G	A	0.0141665	0.00193802	0.592542	2.70E-13
rs2298214	A	C	-0.01241	0.00194099	0.577365	1.60E-10
rs1168114	G	A	0.0155365	0.00199819	0.65237	7.50E-15
rs6693842	C	T	0.013664	0.00199336	0.364844	7.10E-12
rs61748951	A	C	-0.0361204	0.00640878	0.024073	1.70E-08
rs12740811	G	A	-0.0184297	0.00326047	0.094047	1.60E-08
rs2516331	A	C	0.0126835	0.00196658	0.375524	1.10E-10
rs2281718	T	A	0.0596431	0.00195409	0.612575	1.00E-200
rs12740374	T	G	0.0288629	0.00229106	0.221008	2.20E-36
rs140584594	G	A	0.031071	0.00212905	0.729761	3.10E-48
rs267738	G	T	0.0214529	0.00229757	0.219354	9.90E-21
rs12046972	C	T	-0.0148213	0.00191599	0.564083	1.00E-14
rs557933	C	A	0.0152472	0.00190783	0.520349	1.30E-15
rs676210	A	G	0.0592694	0.00235237	0.205227	4.50E-140
rs7583067	T	C	0.0145946	0.00223535	0.240114	6.60E-11
rs17713879	A	G	0.0138956	0.00197564	0.364695	2.00E-12
rs13402475	G	C	-0.0249227	0.00249807	0.815115	1.90E-23
rs2362541	G	T	-0.0108666	0.00190325	0.507519	1.10E-08
rs13389219	T	C	0.0277324	0.00194608	0.392619	4.50E-46
rs2364723	C	G	0.0120956	0.00204641	0.319156	3.40E-09
rs12475332	G	T	0.012897	0.0021638	0.261038	2.50E-09
rs907866	A	G	-0.0183154	0.00191879	0.445487	1.40E-21
rs2723065	G	A	0.0149623	0.00196239	0.376773	2.40E-14
rs4599108	T	C	0.0136654	0.00192434	0.487654	1.20E-12
rs1446585	G	A	0.0167827	0.00217146	0.244295	1.10E-14
rs1083470	A	G	0.0115829	0.00195974	0.615717	3.40E-09
rs12998038	T	C	0.0131918	0.0021847	0.258702	1.60E-09

rs12986742	C	T	-0.0106002	0.00191315	0.475905	3.00E-08
rs6705285	T	G	0.0115225	0.00191933	0.609068	1.90E-09
rs2196808	C	T	0.0128638	0.00215716	0.731961	2.50E-09
rs112350227	T	C	-0.0319379	0.00582274	0.02764	4.10E-08
rs59104589	T	C	0.0150155	0.0019816	0.358286	3.50E-14
rs17326656	T	G	-0.0223923	0.00223885	0.238666	1.50E-23
rs11688682	C	G	0.0147369	0.0022133	0.270836	2.80E-11
rs72926946	A	C	-0.0206565	0.00208041	0.296376	3.10E-23
rs1047891	A	C	-0.0189073	0.00204367	0.315582	2.20E-20
rs78058190	A	G	-0.0783702	0.00490443	0.050415	1.80E-57
rs2943645	T	C	-0.0434706	0.00198833	0.646663	5.90E-106
rs13097947	C	T	0.0159634	0.00203024	0.649315	3.80E-15
rs62246443	C	T	-0.0147841	0.00250895	0.174261	3.80E-09
rs7622114	A	C	0.0115687	0.00193999	0.581017	2.50E-09
rs2159607	T	G	-0.0239454	0.00243213	0.810874	7.20E-23
rs72964564	C	A	-0.0125748	0.00220598	0.250244	1.20E-08
rs6765484	T	C	0.0225207	0.00190864	0.472827	3.90E-32
rs4855582	T	C	0.0111652	0.00192369	0.429621	6.50E-09
rs1225053	C	T	-0.0150376	0.00216684	0.264492	3.90E-12
rs62271373	A	T	-0.0406667	0.00408672	0.059972	2.50E-23
rs13066793	G	A	0.0220769	0.00332887	0.090115	3.30E-11
rs6762415	G	T	-0.0108964	0.00191344	0.535292	1.20E-08
rs77320712	T	G	-0.0127407	0.0022658	0.235452	1.90E-08
rs10513801	G	T	-0.0303325	0.00277034	0.137151	6.70E-28
rs13087167	C	G	0.0167458	0.00198206	0.632997	2.90E-17
rs830620	T	C	0.0149538	0.00193085	0.415386	9.60E-15
rs3732356	T	G	-0.0299026	0.00386524	0.933782	1.00E-14
rs9647335	T	A	0.0277595	0.00242471	0.191928	2.40E-30
rs76247316	C	T	-0.0113866	0.0019083	0.479856	2.40E-09
rs2268840	C	T	0.0173197	0.00226775	0.22846	2.20E-14
rs880674	C	T	0.0149836	0.00273775	0.142056	4.40E-08
rs2237035	T	G	0.0139172	0.00195824	0.385886	1.20E-12
rs1471251	T	A	-0.0193662	0.00195327	0.398159	3.60E-23
rs1383732	G	A	-0.0152024	0.00267698	0.149969	1.40E-08
rs10031010	A	G	0.0138083	0.00246597	0.184617	2.10E-08
rs13137144	A	G	0.0164073	0.00192431	0.461454	1.50E-17
rs71603401	G	A	-0.0154721	0.00279889	0.136851	3.20E-08
rs73243877	G	A	-0.0253693	0.00254909	0.168086	2.50E-23
rs2098918	T	C	0.0118698	0.00191595	0.45513	5.80E-10
rs13111599	G	A	0.0127921	0.00216664	0.737196	3.50E-09
rs1395221	T	G	-0.0111888	0.00195359	0.397646	1.00E-08
rs1055582	T	C	0.0140251	0.00190973	0.505231	2.10E-13

rs13107325	T	C	-0.080346	0.00362445	0.074942	7.00E-109
rs62331150	T	G	-0.0129532	0.00236408	0.205141	4.30E-08
rs13144151	G	A	0.0178695	0.00270341	0.850001	3.80E-11
rs1349852	C	A	0.0112127	0.00192565	0.475293	5.80E-09
rs6824451	A	G	-0.0200444	0.00191167	0.464071	1.00E-25
rs7665587	C	T	0.0139886	0.00193372	0.42208	4.70E-13
rs4691379	T	C	0.0118717	0.00204326	0.318939	6.20E-09
rs10053349	C	T	0.0120071	0.00195336	0.390258	7.90E-10
rs2910949	G	T	0.0133996	0.00199892	0.354888	2.00E-11
rs2307111	C	T	0.0190053	0.0019521	0.394924	2.10E-22
rs2963468	G	A	-0.0196653	0.00226256	0.235297	3.60E-18
rs7725218	A	G	-0.0122469	0.00201433	0.338422	1.20E-09
rs1862205	A	G	0.0113186	0.00194381	0.404725	5.80E-09
rs454968	C	T	0.0110337	0.00199469	0.644812	3.20E-08
rs32578	A	G	0.0133235	0.00206513	0.309015	1.10E-10
rs138354839	A	C	-0.060325	0.00841064	0.015099	7.40E-13
rs116006942	A	G	-0.02987	0.00403491	0.060798	1.30E-13
rs115912456	G	A	0.028084	0.0047884	0.041265	4.50E-09
rs1045241	T	C	0.0164171	0.00215576	0.271252	2.60E-14
rs9327468	A	C	-0.0148994	0.00221907	0.755398	1.90E-11
rs2339234	A	G	-0.0118996	0.00205717	0.683004	7.30E-09
rs188502504	C	T	-0.0316669	0.00510538	0.03643	5.60E-10
rs254562	G	A	-0.0114752	0.00194192	0.409277	3.40E-09
rs2814982	T	C	-0.0275938	0.00314209	0.102216	1.60E-18
rs6939861	A	G	-0.0141972	0.00219368	0.261916	9.70E-11
rs968050	T	C	0.0136601	0.00190807	0.48238	8.10E-13
rs62428831	C	T	0.0178151	0.00275536	0.141547	1.00E-10
rs1281959	G	C	0.0146574	0.00190666	0.525716	1.50E-14
rs1240820	A	G	0.0131835	0.00210043	0.293164	3.50E-10
rs36057735	G	C	-0.0302136	0.00238679	0.199089	1.00E-36
rs635769	C	T	0.0197069	0.00196961	0.628046	1.40E-23
rs41272086	A	G	-0.0567846	0.00310319	0.105505	8.50E-75
rs9465693	A	C	-0.0121004	0.00208231	0.304872	6.20E-09
rs6934962	T	C	0.0161905	0.00194099	0.40151	7.30E-17
rs2750411	G	T	-0.0108405	0.0019091	0.4919	1.40E-08
rs75479205	G	A	0.0138981	0.00243644	0.189496	1.20E-08
rs28746806	C	A	0.0167023	0.00204471	0.333604	3.10E-16
rs147627829	A	G	-0.0535763	0.00468184	0.043706	2.50E-30
rs12205778	A	G	0.0162321	0.00217549	0.258895	8.60E-14
rs998584	A	C	-0.0341901	0.00190975	0.482733	1.10E-71
rs2800710	C	T	-0.0202674	0.00190382	0.51821	1.80E-26
rs9347737	G	A	-0.0134784	0.00194715	0.428295	4.40E-12

rs6460894	C	T	-0.0121653	0.00201452	0.339247	1.60E-09
rs17138358	C	G	-0.0272272	0.00194811	0.398392	2.20E-44
rs201441	G	T	-0.0109672	0.0019342	0.580679	1.40E-08
rs10233430	C	T	-0.0204944	0.00192772	0.427019	2.10E-26
rs55935382	A	C	0.01764	0.00203822	0.32503	4.90E-18
rs73151974	T	C	-0.0161895	0.00273371	0.144024	3.20E-09
rs1534696	A	C	0.0166573	0.0019113	0.540992	2.90E-18
rs2098368	T	C	-0.0116029	0.00192757	0.54591	1.80E-09
rs35493868	G	C	0.0372262	0.00237772	0.202024	3.00E-55
rs183906992	C	T	0.0294539	0.00474735	0.04275	5.50E-10
rs77605964	A	G	0.0170396	0.00227819	0.226924	7.50E-14
rs34940374	A	G	-0.0170378	0.00247025	0.182973	5.30E-12
rs13235365	T	C	0.0258181	0.00214379	0.273616	2.10E-33
rs42125	G	A	-0.0398667	0.0070049	0.023208	1.30E-08
rs12705595	A	G	0.0110554	0.00198306	0.37337	2.50E-08
rs7794796	T	C	-0.016879	0.00203772	0.332335	1.20E-16
rs9987289	G	A	0.0872902	0.00329366	0.908737	9.10E-155
rs75609851	A	G	0.170089	0.00964674	0.010454	1.40E-69
rs140064750	C	T	-0.0440664	0.00634192	0.024546	3.70E-12
rs75662196	C	G	0.0678721	0.00587239	0.027941	6.70E-31
rs61596977	T	C	-0.016225	0.00273721	0.140363	3.10E-09
rs2290866	T	C	-0.0119226	0.00218189	0.253153	4.60E-08
rs7817574	C	T	0.0331985	0.00243942	0.185003	3.50E-42
rs330089	C	T	0.0204692	0.00318925	0.098081	1.40E-10
rs79153732	T	C	-0.0939759	0.00728238	0.017377	4.20E-38
rs10504477	C	T	-0.0150661	0.00193097	0.412192	6.10E-15
rs2247355	T	C	0.0206138	0.00245873	0.182679	5.10E-17
rs6469605	T	C	0.0316197	0.00191959	0.568501	5.80E-61
rs343	A	C	0.133778	0.00346421	0.08307	1.00E-200
rs308	G	T	0.12651	0.00669305	0.020639	1.10E-79
rs13269725	G	A	-0.0259912	0.00353253	0.078342	1.90E-13
rs1431659	G	A	0.0127918	0.00214104	0.728463	2.30E-09
rs4871603	T	C	0.0360061	0.00199228	0.653471	5.20E-73
rs61435086	C	T	0.089343	0.00862169	0.012312	3.70E-25
rs7826177	C	T	0.0112399	0.00197917	0.635428	1.40E-08
rs72647336	A	G	-0.0440494	0.00442834	0.056628	2.60E-23
rs4875043	C	A	-0.0149745	0.0023285	0.216821	1.30E-10
rs10108282	A	T	0.0168525	0.00234262	0.205724	6.30E-13
rs75032664	G	C	-0.0551041	0.00908791	0.012909	1.30E-09
rs142288236	T	C	-0.0776969	0.00795635	0.01469	1.60E-22
rs80005209	G	T	-0.144197	0.00563687	0.029614	2.50E-144
rs4871624	G	T	-0.020416	0.00211032	0.286726	3.90E-22

rs2297409	A	G	-0.0333565	0.00240571	0.194635	1.00E-43
rs1125873	T	A	0.0159741	0.00192237	0.492135	9.60E-17
rs10119644	A	T	0.013468	0.00191042	0.493902	1.80E-12
rs1411432	C	A	-0.0139757	0.00245569	0.186415	1.30E-08
rs74500135	C	T	0.0670758	0.010029	0.009847	2.30E-11
rs1412234	C	T	-0.011989	0.00203583	0.327133	3.90E-09
rs7853377	G	A	0.0152447	0.0023168	0.216224	4.70E-11
rs2417125	G	A	-0.0131524	0.00211658	0.284014	5.20E-10
rs532436	A	G	0.0231547	0.00245365	0.184606	3.80E-21
rs2520096	G	A	0.0147205	0.00215114	0.270192	7.70E-12
rs7036107	G	A	-0.0122524	0.00194377	0.51098	2.90E-10
rs12686780	T	C	-0.0161688	0.00251034	0.174998	1.20E-10
rs2740488	C	A	-0.0686515	0.00216181	0.265328	1.00E-200
rs147772065	C	G	0.0288034	0.0052496	0.034794	4.10E-08
rs686030	A	C	0.0498241	0.00273951	0.858823	6.50E-74
rs2066714	C	T	0.0465396	0.00284369	0.128622	3.40E-60
rs11254464	C	T	0.0127581	0.00193081	0.423107	3.90E-11
rs34045894	A	G	-0.0172909	0.00262706	0.15624	4.60E-11
rs2804894	A	G	0.0173063	0.0021841	0.734831	2.30E-15
rs7924036	T	G	0.0138679	0.00190752	0.503377	3.60E-13
rs703966	A	G	0.0156949	0.00193455	0.418881	4.90E-16
rs1970811	C	T	-0.0116112	0.00191759	0.457743	1.40E-09
rs11009262	T	G	-0.0233289	0.00409245	0.057318	1.20E-08
rs11239536	A	T	0.0287501	0.00223129	0.240967	5.50E-38
rs2068888	A	G	0.0191803	0.0019152	0.450441	1.30E-23
rs10786114	T	C	0.0238735	0.00288231	0.874851	1.20E-16
rs2792751	C	T	-0.0360998	0.0021336	0.725031	3.20E-64
rs12781812	T	G	0.0107203	0.00194289	0.400915	3.40E-08
rs12411732	A	G	-0.0305779	0.00274115	0.145572	6.80E-29
rs61884005	G	C	0.0163406	0.00293002	0.121445	2.40E-08
rs559355	T	A	-0.0349311	0.00261254	0.1573	9.00E-41
rs117847213	G	A	0.0295589	0.0048659	0.040812	1.20E-09
rs59781045	T	C	0.074037	0.0037879	0.068117	4.50E-85
rs17309930	A	C	-0.0218885	0.00235533	0.205132	1.50E-20
rs2302263	T	C	-0.0364373	0.00335268	0.088695	1.60E-27
rs11021232	C	T	-0.0167378	0.00248678	0.18096	1.70E-11
rs964184	C	G	0.10544	0.00279456	0.866364	1.00E-200
rs141469619	G	A	-0.203479	0.0101857	0.009879	8.70E-89
rs4614	G	A	-0.0178217	0.00193829	0.406222	3.80E-20
rs16928809	A	G	-0.0262633	0.00329991	0.093089	1.70E-15
rs12575456	A	G	0.0445866	0.00203342	0.321829	1.40E-106
rs117762989	T	C	-0.0268923	0.00468314	0.043022	9.30E-09

rs4930352	T	G	0.0161709	0.00193852	0.494128	7.30E-17
rs117291242	T	C	-0.0315912	0.00506695	0.036567	4.50E-10
rs79634051	C	G	0.0392148	0.00574877	0.028177	9.00E-12
rs564832	C	T	-0.011708	0.00205455	0.315005	1.20E-08
rs10750766	A	C	-0.0185663	0.00209928	0.709866	9.20E-19
rs2155220	T	C	-0.0105204	0.00191315	0.437951	3.80E-08
rs11218738	A	G	0.0234498	0.00219817	0.249489	1.40E-26
rs174566	G	A	-0.0562241	0.00199551	0.350061	1.20E-174
rs10774439	A	G	0.020513	0.00248318	0.814891	1.40E-16
rs11045171	G	A	0.0283661	0.00240558	0.197605	4.30E-32
rs7316878	C	T	0.0115056	0.00201679	0.43366	1.20E-08
rs2645979	A	G	0.0114281	0.00198606	0.357369	8.70E-09
rs12229011	T	C	-0.0259933	0.00322614	0.097467	7.80E-16
rs7488780	C	G	0.0145459	0.00237104	0.204263	8.50E-10
rs58298943	T	C	0.0201986	0.00342748	0.084318	3.80E-09
rs61352607	T	G	0.0306111	0.00222442	0.240851	4.40E-43
rs3184504	C	T	0.0265387	0.00190452	0.517322	3.90E-44
rs3924313	A	G	-0.0239168	0.00204761	0.322221	1.60E-31
rs58123204	G	A	-0.0184574	0.00264401	0.153445	2.90E-12
rs921919	A	G	-0.041665	0.00206455	0.669162	1.40E-90
rs113740515	A	G	0.0377752	0.00233951	0.209452	1.20E-58
rs11171710	A	G	-0.0114694	0.00192933	0.44814	2.80E-09
rs111363680	T	C	0.0328171	0.00592877	0.031354	3.10E-08
rs9604045	T	G	0.0175151	0.00228914	0.253572	2.00E-14
rs150861794	T	C	-0.0446978	0.00759758	0.018301	4.00E-09
rs76428106	C	T	-0.0624148	0.00878921	0.01297	1.20E-12
rs117230571	G	A	-0.0266417	0.00363105	0.076958	2.20E-13
rs549058	T	G	0.0171095	0.00293694	0.120319	5.70E-09
rs367677	G	A	0.0166703	0.00224786	0.239995	1.20E-13
rs8014289	G	A	0.0151048	0.00192712	0.562282	4.60E-15
rs17124112	A	C	-0.020798	0.00352939	0.07993	3.80E-09
rs1760940	C	A	0.0121791	0.0022157	0.24702	3.90E-08
rs4899251	T	C	-0.0232048	0.0041399	0.942012	2.10E-08
rs7158166	C	T	0.0140932	0.00195101	0.593856	5.10E-13
rs1955512	A	G	0.0109986	0.00197003	0.575802	2.40E-08
rs13379043	C	T	0.0198764	0.00216717	0.28042	4.70E-20
rs2498786	G	C	-0.0254741	0.00196826	0.615979	2.60E-38
rs10162642	A	G	-0.0479223	0.0023419	0.211684	4.60E-93
rs1270076	G	A	0.014345	0.00228637	0.776823	3.50E-10
rs4074448	A	G	0.015131	0.00196355	0.589254	1.30E-14
rs7170463	G	A	0.018911	0.0020591	0.311432	4.20E-20
rs150844304	C	A	-0.090543	0.00599215	0.025907	1.40E-51

rs11631178	C	T	0.0211366	0.0031235	0.103811	1.30E-11
rs28362901	A	C	-0.0238891	0.00336295	0.088332	1.20E-12
rs28510484	C	G	-0.0154472	0.00253932	0.170462	1.20E-09
rs3814883	T	C	-0.0153035	0.00189151	0.482467	5.90E-16
rs4784709	A	T	-0.0747244	0.00477284	0.959159	3.00E-55
rs112233856	G	A	-0.0528133	0.00579493	0.028541	8.00E-20
rs55781197	G	A	0.0587349	0.00295144	0.115034	4.00E-88
rs12928099	A	C	0.0214263	0.00206787	0.29612	3.70E-25
rs113966472	A	G	0.0324769	0.00554864	0.032408	4.80E-09
rs7186799	C	A	-0.0220239	0.00190185	0.437388	5.20E-31
rs71647892	C	T	-0.0629378	0.00871711	0.011842	5.20E-13
rs2925979	C	T	0.037299	0.00205659	0.699999	1.60E-73
rs9933509	C	T	-0.0140121	0.00191313	0.413899	2.40E-13
rs75152587	T	G	-0.0944291	0.00849948	0.012738	1.10E-28
rs9989419	G	A	0.143765	0.00192633	0.605917	1.00E-200
rs140164052	A	G	-0.0417453	0.00540785	0.031786	1.20E-14
rs12926854	G	A	0.0122457	0.00213341	0.269909	9.50E-09
rs11640494	A	G	-0.015349	0.00189969	0.456655	6.50E-16
rs79600951	G	C	-0.106795	0.00325908	0.092057	1.00E-200
rs116857878	T	C	0.0482826	0.00697677	0.020363	4.50E-12
rs9916613	A	T	-0.0133119	0.00199143	0.356493	2.30E-11
rs34138141	T	G	-0.0175318	0.00212461	0.280655	1.60E-16
rs145947882	C	A	-0.164935	0.00609907	0.026042	4.70E-161
rs112001035	A	G	-0.0466304	0.00408312	0.060492	3.30E-30
rs7218647	A	G	0.0109209	0.00192614	0.559041	1.40E-08
rs141062196	A	G	-0.0188553	0.00241089	0.194436	5.20E-15
rs4969141	T	C	0.0296373	0.00191152	0.489676	3.20E-54
rs3027167	T	C	-0.0124258	0.00205083	0.682038	1.40E-09
rs9904004	G	A	-0.0302694	0.00400552	0.060744	4.10E-14
rs2586116	G	C	-0.0162577	0.00217179	0.259435	7.10E-14
rs3794752	C	T	0.0128326	0.00213876	0.280634	2.00E-09
rs8081548	A	T	0.01845	0.00202095	0.658521	6.90E-20
rs56017932	C	G	0.015477	0.00268496	0.20048	8.20E-09
rs73455693	A	G	0.0280646	0.00510538	0.037231	3.90E-08
rs2435307	T	C	0.0162767	0.00190927	0.48634	1.50E-17
rs77960347	G	A	0.290966	0.00830188	0.013349	1.00E-200
rs8086351	G	C	0.0839587	0.0025068	0.824212	1.00E-200
rs11664369	T	C	-0.0229777	0.00215861	0.267061	1.80E-26
rs983663	G	A	-0.0143608	0.0022075	0.251682	7.70E-11
rs150237291	C	T	0.0453058	0.00656295	0.021799	5.10E-12
rs680321	C	T	0.0108517	0.00191658	0.456556	1.50E-08
rs2298624	T	C	0.0300361	0.00280916	0.132532	1.10E-26

rs3745683	A	G	-0.0546353	0.00362928	0.074638	3.20E-51
rs429358	C	T	-0.0756554	0.00263871	0.154325	8.71E-181
rs12462109	T	C	-0.0159539	0.00210812	0.287067	3.80E-14
rs367070	G	A	0.0417949	0.00228709	0.225571	1.30E-74
rs554146	C	A	0.0127257	0.00211531	0.704992	1.80E-09
rs62117487	G	A	0.0457487	0.00411744	0.057038	1.10E-28
rs116843064	A	G	0.206178	0.0069176	0.019327	3.40E-195
rs62102718	T	A	-0.0236953	0.0021125	0.286413	3.40E-29
rs4803773	G	A	0.0400846	0.00199508	0.498722	8.70E-90
rs7251640	C	T	0.0141902	0.00242811	0.194019	5.10E-09
rs2965169	C	A	0.0120831	0.00195448	0.388738	6.30E-10
rs144311893	T	C	0.0809615	0.0066916	0.022086	1.10E-33
rs1132274	A	C	-0.0217809	0.00263979	0.154029	1.60E-16
rs6018652	A	G	0.0259271	0.0023605	0.792888	4.60E-28
rs6123685	A	G	0.0159783	0.0021911	0.254745	3.00E-13
rs144033177	C	A	-0.05557	0.0078389	0.015822	1.40E-12
rs150224153	T	C	-0.0934689	0.00575879	0.029665	3.10E-59
rs77767539	A	G	0.0452639	0.00821382	0.014299	3.60E-08
rs6066148	C	G	0.0133124	0.00218121	0.259964	1.00E-09
rs6059958	T	C	0.0146541	0.00255516	0.170742	9.70E-09
rs6142206	A	G	-0.0161106	0.00193468	0.42072	8.30E-17
rs6073958	C	T	-0.0609299	0.00239207	0.198804	4.10E-143
rs6075860	A	G	-0.0128	0.00192763	0.56549	3.10E-11
rs76602912	C	T	-0.0434476	0.00626364	0.023789	4.00E-12
rs407133	C	G	-0.0105618	0.00193239	0.558791	4.60E-08
rs235314	T	C	-0.0177425	0.00191993	0.532212	2.40E-20
rs7281183	A	G	-0.01272	0.00218963	0.737142	6.30E-09
rs2236464	C	T	-0.0155836	0.00235595	0.212102	3.70E-11
rs3746915	G	A	0.0108813	0.00194098	0.580939	2.10E-08
rs2256609	G	A	-0.0328881	0.00244058	0.189238	2.20E-41
rs133015	G	C	0.0205993	0.00192854	0.440063	1.20E-26
rs9622830	G	C	-0.0163821	0.00199965	0.354212	2.60E-16
rs460428	C	T	-0.0143462	0.00226779	0.230673	2.50E-10

A1: Effect Allele; A2: Alternative Allele; EAF: Effect Allele Frequency; Beta: beta coefficient; SE: Standard error of beta coefficient; *p*: *p*-value of the meta-analysis

Table S2. SNPs of LDL-C extracted from UK Biobank with statistically significant threshold

rsID	A1	A2	BETA	SE	EAF	*p* value
rs115458560	C	T	-0.0503391	0.0077049	0.018563	6.40E-11
rs6667939	T	C	0.0153061	0.00232969	0.71867	5.00E-11
rs12078100	G	C	0.0131127	0.00214876	0.623087	1.00E-09
rs114165349	C	G	0.056229	0.00695521	0.023172	6.20E-16

rs140584594	G	A	-0.01433	0.00232961	0.730069	7.70E-10
rs1183851	C	T	0.024377	0.00213943	0.395544	4.50E-30
rs6680227	A	G	-0.0746581	0.00563322	0.035237	4.30E-40
rs2642438	G	A	0.0253218	0.00227308	0.702657	8.00E-29
rs28631087	C	T	-0.0161901	0.0025459	0.21274	2.00E-10
rs880315	C	T	-0.0152225	0.00220678	0.34024	5.30E-12
rs55637835	T	C	-0.0187129	0.00324835	0.120881	8.40E-09
rs11591147	T	G	-0.348456	0.00793088	0.017468	1.00E-200
rs472495	T	G	0.0425743	0.00218093	0.648959	7.30E-85
rs4970834	T	C	-0.105303	0.00268179	0.186642	1.00E-200
rs556107	T	C	0.0351712	0.00208846	0.523338	1.20E-63
rs1556562	T	G	0.0190249	0.00248903	0.790033	2.10E-14
rs10910476	T	C	0.0123938	0.00210193	0.555163	3.70E-09
rs12471768	C	T	0.0135999	0.00228112	0.704119	2.50E-09
rs6732741	A	T	-0.0280985	0.00322423	0.119324	2.90E-18
rs6709904	G	A	-0.043441	0.00329601	0.112585	1.10E-39
rs4666384	G	A	-0.016633	0.00224003	0.678914	1.10E-13
rs934197	A	G	0.0832348	0.00220298	0.33544	1.00E-200
rs7562734	C	G	-0.0201126	0.00222879	0.322299	1.80E-19
rs150474434	A	G	-0.0347623	0.00347063	0.101353	1.30E-23
rs17050272	A	G	-0.0205597	0.00211549	0.409275	2.50E-22
rs7569317	C	T	0.017881	0.00208232	0.530638	8.90E-18
rs56236159	G	T	0.0177139	0.00307979	0.13149	8.80E-09
rs4954192	T	C	0.0146629	0.00213654	0.373474	6.70E-12
rs2287622	G	A	-0.0211572	0.00212931	0.602794	2.90E-23
rs1250258	T	C	0.0137745	0.00236771	0.736649	6.00E-09
rs11568318	A	C	0.0256951	0.00417849	0.066246	7.80E-10
rs1260326	C	T	-0.0347299	0.00212546	0.604443	5.10E-60
rs6544713	C	T	-0.0537152	0.00222274	0.676864	5.00E-129
rs13076933	G	T	-0.020994	0.00239027	0.259454	1.60E-18
rs9832727	G	C	-0.0147604	0.00220388	0.339636	2.10E-11
rs9834932	G	A	-0.0322172	0.00366408	0.08883	1.50E-18
rs71311871	G	A	-0.0283073	0.00378458	0.083052	7.50E-14
rs9289196	C	T	0.0173702	0.00275673	0.173786	3.00E-10
rs113177823	A	G	-0.0411737	0.00466039	0.054136	1.00E-18
rs3732359	A	G	-0.0173556	0.00252038	0.78012	5.70E-12
rs13121616	G	A	0.0128507	0.00226135	0.693553	1.30E-08
rs9884390	C	T	0.0251489	0.00249163	0.234121	5.90E-24
rs1229984	C	T	0.0525797	0.00633356	0.972799	1.00E-16
rs990619	G	C	-0.0118015	0.00208525	0.523638	1.50E-08
rs13108218	G	A	-0.0177871	0.0021598	0.614867	1.80E-16
rs11099097	T	C	-0.0181876	0.00230231	0.291333	2.80E-15

rs13107325	T	C	-0.0248331	0.00396439	0.074895	3.80E-10
rs12916	C	T	0.0621175	0.00212705	0.400537	1.70E-187
rs146433259	T	C	-0.0569699	0.0103828	0.011736	4.10E-08
rs28590710	A	G	-0.017618	0.00274759	0.196791	1.40E-10
rs7734476	A	G	0.0187595	0.00209401	0.550077	3.30E-19
rs6874202	C	T	0.032331	0.00216292	0.634269	1.60E-50
rs116734477	T	C	-0.0473368	0.0052819	0.040837	3.20E-19
rs3104412	G	A	-0.0189061	0.00209423	0.451531	1.80E-19
rs2745353	T	C	0.0126963	0.00208336	0.5181	1.10E-09
rs7776054	G	A	-0.0162316	0.00237493	0.261313	8.20E-12
rs12208357	T	C	0.0569843	0.00409617	0.070102	5.40E-44
rs146534110	T	G	0.0680211	0.00910045	0.013304	7.80E-14
rs118039278	A	G	0.0835056	0.00388533	0.078672	1.80E-102
rs7746081	A	G	-0.0234725	0.00226701	0.304113	4.00E-25
rs9496567	A	G	-0.0174316	0.00243272	0.243458	7.80E-13
rs79220007	C	T	-0.057338	0.00392142	0.076135	2.00E-48
rs3823376	T	C	0.0172774	0.0020812	0.502179	1.00E-16
rs34568880	T	C	0.0560827	0.00908804	0.013313	6.80E-10
rs3822855	T	G	0.0178418	0.00212447	0.40166	4.50E-17
rs117733303	G	A	0.0838178	0.00773844	0.018422	2.40E-27
rs2820226	A	G	-0.012864	0.00209731	0.451903	8.60E-10
rs9471968	G	A	-0.0116148	0.00209345	0.545696	2.90E-08
rs869412	C	T	-0.0142314	0.00250093	0.225684	1.30E-08
rs28406917	T	C	0.0119057	0.00211433	0.427624	1.80E-08
rs56130071	C	G	0.0331798	0.00253796	0.216984	4.70E-39
rs61003864	C	T	0.0155966	0.00267616	0.187128	5.60E-09
rs60612724	G	A	0.0323852	0.00541912	0.038647	2.30E-09
rs111338114	G	A	-0.0287743	0.00492283	0.05601	5.10E-09
rs10231941	C	T	0.0198565	0.00272709	0.177717	3.30E-13
rs4148826	C	T	-0.0157917	0.0027233	0.18045	6.70E-09
rs2073547	G	A	0.0355498	0.00267287	0.184007	2.30E-40
rs117139027	A	G	-0.0571106	0.00792993	0.017594	5.90E-13
rs28615248	C	T	0.0184347	0.00263568	0.196095	2.70E-12
rs4738684	G	A	-0.0315439	0.00220937	0.664652	3.00E-46
rs55831924	T	C	0.0156761	0.0021829	0.361448	6.90E-13
rs1350559	G	C	0.0137824	0.00213685	0.401347	1.10E-10
rs2737265	G	A	-0.0202743	0.002322	0.28046	2.50E-18
rs28601761	G	C	-0.0620105	0.00213624	0.418688	2.90E-185
rs9987289	G	A	0.0453824	0.00362169	0.908808	5.10E-36
rs34265667	A	G	-0.0320529	0.00575731	0.033987	2.60E-08
rs28814720	G	A	0.0121747	0.00215073	0.519766	1.50E-08
rs10448340	G	T	-0.0150594	0.00223737	0.319765	1.70E-11

rs2519093	T	C	0.0557289	0.0026865	0.18451	1.40E-95
rs3780181	G	A	-0.0278983	0.00418323	0.067627	2.60E-11
rs6475606	T	C	-0.0202689	0.00208367	0.48447	2.30E-22
rs6560499	A	G	-0.0122028	0.00212189	0.576007	8.90E-09
rs2066714	C	T	0.0210151	0.00310964	0.128775	1.40E-11
rs2740488	C	A	-0.0252452	0.00236503	0.265466	1.30E-26
rs79828839	T	C	0.0144987	0.00261498	0.198784	2.90E-08
rs2068888	A	G	-0.0192081	0.00209421	0.450695	4.60E-20
rs76468627	T	C	-0.0217346	0.00394926	0.07587	3.70E-08
rs2250802	A	G	-0.0181939	0.00233224	0.72434	6.10E-15
rs11014204	T	C	0.0137924	0.00233007	0.280227	3.20E-09
rs17476364	C	T	-0.0216418	0.00335937	0.108328	1.20E-10
rs12246352	G	A	0.0257335	0.00342791	0.103895	6.00E-14
rs11601507	A	C	0.0322012	0.00404981	0.069468	1.80E-15
rs11226108	C	G	-0.016026	0.00265376	0.191562	1.60E-09
rs10832963	G	T	0.0173059	0.00239616	0.744224	5.10E-13
rs174564	G	A	-0.0318977	0.0021896	0.348557	4.50E-48
rs964184	C	G	-0.0575823	0.00306356	0.866409	8.20E-79
rs7108486	C	T	-0.0386029	0.00692024	0.023721	2.40E-08
rs4930163	A	G	0.0174597	0.00285203	0.159456	9.20E-10
rs4307732	A	G	0.0448315	0.0033985	0.105876	9.80E-40
rs2160994	C	T	0.0182581	0.00218417	0.647494	6.30E-17
rs61754230	T	C	0.0430117	0.00748441	0.019784	9.10E-09
rs597808	G	A	0.0271066	0.00209151	0.515915	2.10E-38
rs35882350	G	A	0.0139684	0.00237417	0.261067	4.00E-09
rs11065385	G	A	-0.0244904	0.00225744	0.690891	2.00E-27
rs2391825	A	G	-0.0129848	0.00233356	0.279219	2.60E-08
rs2238162	T	C	-0.0165008	0.00208847	0.522955	2.80E-15
rs6602912	G	T	0.0221997	0.00231001	0.284854	7.20E-22
rs11621792	T	C	0.0191489	0.00211033	0.452867	1.10E-19
rs61988556	C	T	-0.0224158	0.00372109	0.086142	1.70E-09
rs145730801	C	T	0.0359995	0.00516918	0.044378	3.30E-12
rs2043085	C	T	-0.017018	0.00214177	0.612225	1.90E-15
rs6495122	C	A	0.0143852	0.00211976	0.591279	1.20E-11
rs12445804	A	G	0.0229888	0.0040052	0.074901	9.50E-09
rs7202323	G	T	-0.0255365	0.00247732	0.229741	6.50E-25
rs4782568	G	C	-0.0165059	0.00210805	0.450902	4.90E-15
rs183130	T	C	-0.0329541	0.00223034	0.323808	2.10E-49
rs62033400	G	A	-0.0144167	0.00213447	0.394662	1.40E-11
rs9929977	A	T	0.0167269	0.00216129	0.369846	1.00E-14
rs34042070	G	C	0.0485227	0.00268011	0.187927	2.90E-73
rs72631343	G	C	-0.0292893	0.00311032	0.128894	4.60E-21

rs1801689	C	A	0.061862	0.00605788	0.030551	1.80E-24
rs72911393	T	C	-0.0182619	0.00294119	0.147618	5.30E-10
rs55714927	T	C	-0.0264314	0.00265551	0.190364	2.40E-23
rs9894946	G	A	-0.0173333	0.00290132	0.841112	2.30E-09
rs77542162	G	A	0.128484	0.0070401	0.022495	2.10E-74
rs77960347	G	A	0.0706209	0.00908667	0.013351	7.70E-15
rs7241918	T	G	0.0157471	0.00274939	0.82411	1.00E-08
rs440677	A	G	-0.0159238	0.00215434	0.623063	1.50E-13
rs35511051	A	C	-0.0217862	0.00254546	0.209985	1.10E-17
rs2738447	C	A	0.0422734	0.00211024	0.592612	2.90E-89
rs1551891	A	G	-0.173628	0.00366029	0.088107	1.00E-200
rs4263041	G	A	-0.0699797	0.00251019	0.283209	4.90E-171
rs111273322	A	G	0.0217698	0.00334926	0.108352	8.00E-11
rs5112	G	C	0.0277824	0.00222587	0.533387	9.40E-36
rs516316	C	G	0.0296183	0.00206761	0.507935	1.50E-46
rs143020224	G	C	-0.168952	0.00320833	0.118603	1.00E-200
rs8107974	T	A	-0.105058	0.00391919	0.075765	2.70E-158
rs375972689	G	T	0.168281	0.0101662	0.01069	1.50E-61
rs1010759	A	G	-0.0231042	0.00300476	0.140367	1.50E-14
rs1883711	C	G	0.10277	0.0060919	0.031412	7.50E-64
rs6031587	T	C	-0.0256479	0.00413761	0.071963	5.70E-10
rs6074012	C	T	0.0116537	0.00208962	0.523914	2.40E-08
rs17569873	T	C	0.0171196	0.00260335	0.201299	4.80E-11
rs438568	G	A	0.0124777	0.00213986	0.609246	5.50E-09
rs2618566	T	G	-0.0249198	0.00219993	0.659861	9.60E-30
rs2256814	A	G	0.0152618	0.00262512	0.197877	6.10E-09
rs960596	T	C	0.0135068	0.00221758	0.33932	1.10E-09
rs12162782	G	T	0.0129592	0.00219352	0.344365	3.50E-09

A1: Effect Allele; A2: Alternative Allele; EAF: Effect Allele Frequency; Beta: beta coefficient; SE: Standard error of beta coefficient; *p*: *p*-value of the meta-analysis

Table S3. SNPs of triglycerides extracted from UK Biobank with statistically significant threshold

rsID	A1	A2	BETA	SE	EAF	*p* value
rs11206374	A	G	0.0250232	0.00237577	0.224815	6.10E-26
rs74090351	A	G	-0.0250097	0.00393658	0.068143	2.10E-10
rs9436661	G	T	-0.0777322	0.00207793	0.352919	1.00E-200
rs1044808	C	G	-0.0247492	0.00362188	0.081237	8.30E-12
rs1760801	A	G	-0.0202981	0.00218442	0.295768	1.50E-20
rs9425589	A	G	-0.0137618	0.00200182	0.566781	6.20E-12
rs114165349	C	G	0.0818126	0.00663594	0.023157	6.30E-35
rs213494	T	C	0.0156106	0.00207578	0.648345	5.50E-14

rs698927	C	A	-0.0183059	0.00256302	0.183785	9.20E-13
rs320369	G	A	-0.0125327	0.00214567	0.683292	5.20E-09
rs880315	C	T	-0.011772	0.00210497	0.340211	2.20E-08
rs36043408	A	G	-0.0128072	0.00198554	0.502153	1.10E-10
rs61830291	C	A	0.0287484	0.00337913	0.095791	1.80E-17
rs11122450	G	T	-0.0481745	0.0020373	0.611733	1.30E-123
rs2131311	G	A	-0.0124181	0.00222753	0.714636	2.50E-08
rs1938566	T	C	-0.0212127	0.00267054	0.834537	2.00E-15
rs11240358	A	G	0.0135651	0.00202953	0.394026	2.30E-11
rs11118310	T	A	0.0193178	0.00202011	0.593476	1.10E-21
rs1043897	T	G	-0.014664	0.00201517	0.415585	3.40E-13
rs1473886	T	G	-0.0181218	0.00198519	0.477624	6.90E-20
rs954244	G	C	0.015323	0.00227424	0.254641	1.60E-11
rs2382825	T	C	-0.0134774	0.00204919	0.622954	4.80E-11
rs676210	A	G	-0.0735169	0.00244968	0.205293	7.10E-198
rs4665972	C	T	-0.10025	0.00203577	0.604587	1.00E-200
rs150419156	A	G	-0.0505491	0.00847063	0.015259	2.40E-09
rs10210970	T	C	0.0232557	0.00292788	0.131448	2.00E-15
rs13389219	T	C	-0.0376242	0.00202704	0.392551	6.60E-77
rs3731696	G	A	0.0218939	0.00303176	0.121188	5.10E-13
rs78058190	A	G	0.0816071	0.00510649	0.05045	1.70E-57
rs2943645	T	C	0.0402918	0.00207151	0.646594	2.90E-84
rs12475332	G	T	-0.0140007	0.00225386	0.260988	5.20E-10
rs3820897	C	T	0.0197269	0.00260304	0.819861	3.50E-14
rs72784786	A	G	0.0263468	0.00416714	0.060638	2.60E-10
rs6752845	C	G	-0.0137812	0.00200389	0.431425	6.10E-12
rs7424120	T	C	-0.0123561	0.00203065	0.601724	1.20E-09
rs1009360	C	T	-0.0184832	0.00200785	0.41859	3.40E-20
rs1420384	T	G	-0.01279	0.00210534	0.66699	1.20E-09
rs11904650	G	A	0.0410641	0.00692419	0.020999	3.00E-09
rs4128205	C	A	0.0115011	0.00199472	0.509173	8.10E-09
rs17326656	T	G	0.0174394	0.00233365	0.238472	7.80E-14
rs62135012	A	G	-0.0117482	0.00207507	0.357929	1.50E-08
rs6432622	G	A	-0.0108868	0.00198007	0.490264	3.80E-08
rs4675812	A	G	-0.0142305	0.00201176	0.587882	1.50E-12
rs6800707	G	C	0.0298848	0.00253832	0.810543	5.30E-32
rs62271373	A	T	0.0419639	0.00426588	0.059967	7.80E-23
rs62274099	T	C	0.0120882	0.00202424	0.424205	2.30E-09
rs2137557	C	T	0.0117624	0.00208003	0.645612	1.60E-08
rs6805924	T	G	0.0109684	0.00200571	0.430548	4.50E-08
rs684773	C	A	0.0291133	0.00234718	0.766797	2.50E-35
rs3103310	G	A	0.0202549	0.00236652	0.241785	1.10E-17

rs9831084	C	T	-0.0118516	0.00199656	0.461922	2.90E-09
rs73238173	G	C	-0.0165938	0.00295427	0.129955	1.90E-08
rs10513688	A	G	0.0247828	0.00334924	0.097403	1.40E-13
rs79287178	A	G	0.0500946	0.00600435	0.031151	7.20E-17
rs11185542	C	G	-0.0126395	0.00223848	0.727914	1.60E-08
rs6792725	G	A	-0.0152543	0.00221886	0.692404	6.20E-12
rs13066793	G	A	-0.0224583	0.00347278	0.090095	1.00E-10
rs12504746	T	C	-0.0152674	0.00252148	0.192857	1.40E-09
rs2237029	A	G	-0.0139399	0.00204019	0.601353	8.30E-12
rs13101504	G	T	0.0195598	0.00240753	0.412851	4.50E-16
rs1347188	G	A	0.0139021	0.00231385	0.245797	1.90E-09
rs71603401	G	A	0.0264991	0.00291753	0.136804	1.10E-19
rs11722924	C	G	0.0129095	0.00199093	0.53531	8.90E-11
rs13118477	A	G	0.0149897	0.00203847	0.392654	1.90E-13
rs148827772	G	A	0.0465516	0.00710636	0.022544	5.70E-11
rs11100083	C	T	-0.0160248	0.00237585	0.226214	1.50E-11
rs278981	C	T	0.0125871	0.00229058	0.758155	3.90E-08
rs3775228	T	C	0.0338197	0.00203602	0.399717	5.80E-62
rs6532798	T	C	0.013792	0.00216353	0.697405	1.80E-10
rs13108218	G	A	-0.0305169	0.00205811	0.614859	9.70E-50
rs13107325	T	C	0.0300749	0.00377743	0.074905	1.70E-15
rs7735249	G	C	0.0268635	0.00315997	0.112767	1.90E-17
rs55646464	T	G	0.0121913	0.00217016	0.300087	1.90E-08
rs72644085	C	T	-0.0196036	0.00281241	0.146036	3.20E-12
rs193735	A	G	0.0329807	0.00529922	0.036665	4.90E-10
rs970069	T	C	0.0162322	0.00243455	0.212039	2.60E-11
rs6882076	C	T	0.0331011	0.00206122	0.634285	4.90E-58
rs62397245	G	C	0.0149941	0.00239634	0.22236	3.90E-10
rs11746801	A	G	-0.0123657	0.00207937	0.637069	2.70E-09
rs4976033	G	A	0.0177909	0.00205178	0.402122	4.30E-18
rs7704653	G	A	0.0157533	0.00224582	0.723029	2.30E-12
rs72801474	A	G	-0.0307938	0.00344154	0.091843	3.60E-19
rs13354321	C	T	-0.0154305	0.00201429	0.409787	1.90E-14
rs1316753	C	G	-0.0145732	0.00202974	0.394062	7.00E-13
rs7714361	C	A	0.0138858	0.00236622	0.233667	4.40E-09
rs1045241	T	C	-0.0206988	0.00224699	0.270943	3.20E-20
rs325485	G	A	-0.0117435	0.00204042	0.602889	8.60E-09
rs7244	A	G	0.0151978	0.00261808	0.174166	6.40E-09
rs78588343	A	G	-0.0155231	0.0026035	0.176216	2.50E-09
rs28752924	G	T	0.0243875	0.00276364	0.243148	1.10E-18
rs186413375	C	A	0.037979	0.00423249	0.083486	2.90E-19
rs138191773	A	G	-0.0471717	0.00791884	0.017021	2.60E-09

rs17585887	C	T	-0.0285849	0.00201656	0.591074	1.30E-45
rs9376511	G	A	-0.0154993	0.00246511	0.203288	3.20E-10
rs4134963	T	C	-0.0189544	0.00254235	0.189878	9.00E-14
rs28383314	C	T	0.0379005	0.00204671	0.624109	1.50E-76
rs77009508	G	A	0.0450912	0.00379997	0.073734	1.80E-32
rs9368503	T	A	-0.0119616	0.00198801	0.510113	1.80E-09
rs998584	A	C	0.0401182	0.00198999	0.482727	2.20E-90
rs62427982	T	C	-0.0133153	0.00213196	0.32217	4.20E-10
rs186696265	T	C	-0.104318	0.00828522	0.014693	2.40E-36
rs729761	G	T	0.0177868	0.00221435	0.711859	9.50E-16
rs1835346	G	A	-0.0391877	0.00654659	0.023874	2.20E-09
rs1064173	A	G	-0.0213585	0.00230298	0.284399	1.80E-20
rs2983896	A	G	0.0137912	0.00242177	0.21474	1.20E-08
rs9480889	G	C	0.0163067	0.0024101	0.783137	1.30E-11
rs6916318	T	A	0.0267283	0.0019907	0.530533	4.20E-41
rs73025562	A	G	0.0138633	0.00231292	0.245879	2.00E-09
rs852388	C	G	0.0157124	0.00245601	0.211278	1.60E-10
rs62459095	T	C	-0.0319865	0.00426882	0.061781	6.70E-14
rs2240466	A	G	-0.122769	0.00301848	0.122916	1.00E-200
rs41785	A	C	-0.0150445	0.00201492	0.417487	8.20E-14
rs62473520	C	T	-0.0211003	0.00380118	0.077998	2.80E-08
rs71538127	G	C	0.0176494	0.00303718	0.122052	6.20E-09
rs10242866	T	C	0.015793	0.00202877	0.398698	7.00E-15
rs1799831	T	C	0.024506	0.00273953	0.156029	3.70E-19
rs7786339	T	C	0.016328	0.00265352	0.168936	7.60E-10
rs72555385	G	A	0.0653776	0.0046178	0.048975	1.70E-45
rs10277582	T	C	-0.0173714	0.00308015	0.118591	1.70E-08
rs2699805	A	G	-0.0201313	0.00203748	0.399672	5.10E-23
rs12669911	C	A	-0.0118536	0.00205405	0.614026	7.90E-09
rs72603744	C	A	-0.0125232	0.00213684	0.315623	4.60E-09
rs12530679	G	A	-0.0121299	0.00201682	0.48466	1.80E-09
rs75721796	A	G	0.0210491	0.00245993	0.214459	1.20E-17
rs4731701	T	C	-0.0325803	0.00198921	0.493088	2.70E-60
rs4841580	C	T	-0.0245383	0.00200093	0.434941	1.40E-34
rs79153732	T	C	0.0773626	0.00758596	0.017389	2.00E-24
rs7000494	C	G	0.136538	0.00584159	0.030042	8.00E-121
rs150564454	A	G	-0.101751	0.00963178	0.011556	4.40E-26
rs1544980	C	T	0.0238704	0.00248513	0.198644	7.60E-22
rs343	A	C	-0.141493	0.00361052	0.083129	1.00E-200
rs2081687	C	T	-0.0261379	0.00209126	0.663201	7.60E-36
rs6999569	G	A	-0.0860885	0.00198061	0.470737	1.00E-200
rs13264304	G	C	0.0189102	0.00276507	0.151023	8.00E-12

rs308	G	T	-0.159413	0.00698442	0.020597	2.60E-115
rs13269725	G	A	0.0349878	0.0036804	0.078416	2.00E-21
rs3808477	T	C	-0.0133854	0.00220342	0.279294	1.20E-09
rs75609851	A	G	-0.199116	0.0100401	0.01048	1.60E-87
rs2407278	G	A	-0.0339449	0.00581961	0.030154	5.40E-09
rs28439112	A	T	0.0126014	0.00226754	0.255528	2.70E-08
rs2244278	A	C	-0.0269912	0.00306133	0.120553	1.20E-18
rs1567353	G	C	0.0148202	0.00216358	0.307644	7.40E-12
rs581080	C	G	0.0175432	0.00258442	0.819008	1.10E-11
rs696825	T	C	-0.0202838	0.00228763	0.252926	7.50E-19
rs10797119	C	T	0.0157113	0.00200465	0.536622	4.60E-15
rs2131919	G	A	0.0173511	0.00268396	0.164266	1.00E-10
rs2519093	T	C	-0.021158	0.00256189	0.184548	1.50E-16
rs7847285	C	T	-0.0116104	0.00202626	0.589213	1.00E-08
rs10811662	A	G	-0.0154098	0.0026291	0.173212	4.60E-09
rs7861679	T	C	0.0122025	0.00216741	0.696815	1.80E-08
rs11187019	G	A	-0.0116437	0.00200546	0.550621	6.40E-09
rs10822163	G	C	-0.0321674	0.00199059	0.473369	9.70E-59
rs7077812	C	T	0.0142284	0.00250611	0.194911	1.40E-08
rs2068888	A	G	-0.0318216	0.00199589	0.450732	3.20E-57
rs56397607	G	A	0.0174851	0.00257608	0.182538	1.10E-11
rs80276949	A	G	0.0457563	0.00669008	0.022677	8.00E-12
rs113344423	A	G	0.0434468	0.00428926	0.060177	4.10E-24
rs55767272	C	A	-0.0282011	0.00403602	0.065077	2.80E-12
rs11000468	T	C	-0.0148544	0.00231748	0.255269	1.50E-10
rs2487294	T	G	0.0183191	0.00222084	0.723477	1.60E-16
rs140107293	G	A	-0.0227003	0.00275296	0.154707	1.60E-16
rs3758413	C	T	0.0111232	0.00201767	0.417724	3.50E-08
rs10883026	T	C	-0.0144604	0.00200472	0.521569	5.50E-13
rs2773469	G	A	-0.0188717	0.00225426	0.733286	5.70E-17
rs1133400	G	A	0.0137462	0.00239759	0.219923	9.80E-09
rs499293	A	G	-0.0117912	0.00208902	0.65807	1.70E-08
rs174566	G	A	0.0485019	0.00207982	0.34974	2.80E-120
rs480823	C	T	0.15572	0.00371974	0.078996	1.00E-200
rs150423652	T	G	0.285824	0.0122924	0.006675	1.40E-119
rs551243	C	G	0.0143952	0.00199178	0.469258	4.90E-13
rs10899490	T	C	-0.0170082	0.00269347	0.161369	2.70E-10
rs61905078	C	A	0.199898	0.00379494	0.073841	1.00E-200
rs150555490	T	C	-0.0384234	0.00427544	0.056868	2.50E-19
rs61729990	A	C	-0.0558488	0.00752135	0.017888	1.10E-13
rs117291242	T	C	0.0297851	0.00528225	0.036536	1.70E-08
rs117287238	A	G	-0.0394324	0.00614684	0.028185	1.40E-10

rs75268115	G	A	-0.0207435	0.0035798	0.084699	6.80E-09
rs326222	C	T	0.0252203	0.00215698	0.697957	1.40E-31
rs11600815	A	G	-0.0322469	0.00458644	0.051753	2.10E-12
rs2302263	T	C	0.0436112	0.00349051	0.088863	8.00E-36
rs7947951	G	A	0.0193777	0.00214074	0.689254	1.40E-19
rs11030107	G	A	0.0160418	0.00225693	0.260757	1.20E-12
rs3974807	T	C	0.0159776	0.00253125	0.189267	2.80E-10
rs10750766	A	C	0.0193855	0.00218742	0.709959	7.80E-19
rs78484485	A	G	-0.0758529	0.00439863	0.054016	1.20E-66
rs79357714	G	A	-0.0289463	0.00476453	0.045724	1.20E-09
rs75942983	T	A	-0.0201303	0.00361206	0.083627	2.50E-08
rs10773049	C	T	-0.0290398	0.00203691	0.39535	4.10E-46
rs67981690	G	A	0.0299225	0.00297454	0.129887	8.30E-24
rs4760254	C	G	-0.0281414	0.00232488	0.23908	1.00E-33
rs139386986	T	C	-0.0216903	0.0035608	0.092129	1.10E-09
rs4761234	C	T	-0.0140236	0.00199376	0.484365	2.00E-12
rs117233107	A	G	-0.0732317	0.0086122	0.015176	1.80E-17
rs10773000	T	G	-0.0149294	0.0021148	0.332276	1.70E-12
rs7308584	A	G	0.0149992	0.00256911	0.184436	5.30E-09
rs7134375	A	C	-0.0171435	0.00200504	0.431174	1.20E-17
rs7135509	C	T	-0.0121338	0.00221197	0.293196	4.10E-08
rs35763453	C	T	0.02845	0.00435137	0.058647	6.20E-11
rs12424054	A	G	0.0191212	0.00235346	0.232437	4.50E-16
rs4765148	T	G	-0.0251537	0.0021456	0.312686	9.70E-32
rs149778057	C	A	-0.0157568	0.0022259	0.32832	1.50E-12
rs7400002	G	A	0.0139701	0.00236663	0.230609	3.60E-09
rs7140110	C	T	0.0283172	0.00217811	0.298338	1.20E-38
rs6562773	G	A	-0.0120538	0.00201057	0.547533	2.00E-09
rs9561643	C	A	0.0167149	0.00214481	0.314726	6.50E-15
rs1340819	C	A	-0.0121655	0.00209472	0.345011	6.30E-09
rs2812208	C	G	-0.048426	0.00696323	0.02089	3.50E-12
rs9584870	C	T	-0.0123562	0.00210377	0.366179	4.30E-09
rs2070341	T	C	0.0113012	0.00203413	0.60309	2.80E-08
rs2240533	C	T	-0.0129897	0.00215234	0.309898	1.60E-09
rs61993685	C	T	-0.0234087	0.00373997	0.076413	3.90E-10
rs6572807	G	A	0.0124759	0.00224899	0.267065	2.90E-08
rs12880341	C	T	0.0209696	0.00273647	0.159023	1.80E-14
rs56902258	A	T	-0.0151434	0.0025129	0.196198	1.70E-09
rs139974673	C	T	0.143073	0.00623867	0.026131	2.20E-116
rs11637681	G	A	0.0125866	0.00224251	0.276291	2.00E-08
rs150460588	C	T	0.0326952	0.00484966	0.044425	1.60E-11
rs2043085	C	T	-0.0308028	0.00204157	0.612184	1.90E-51

rs1077835	G	A	0.0473668	0.00240452	0.220459	2.20E-86
rs12440800	T	A	0.0161	0.00229294	0.255149	2.20E-12
rs12902047	C	A	-0.0129253	0.00214533	0.313907	1.70E-09
rs10152471	A	G	-0.0135209	0.00204778	0.388656	4.00E-11
rs275184	G	T	-0.0173487	0.00275263	0.162051	2.90E-10
rs17184382	C	A	-0.0219251	0.00201039	0.425367	1.10E-27
rs1037117	A	G	0.0172213	0.00229153	0.254798	5.70E-14
rs143076454	A	G	0.0403999	0.00735364	0.018647	3.90E-08
rs12926107	G	A	0.0126364	0.0020014	0.454903	2.70E-10
rs2937124	T	C	-0.0182561	0.00212336	0.362769	8.10E-18
rs12928099	A	C	-0.0282116	0.00218133	0.296442	2.90E-38
rs3814883	T	C	0.0148926	0.00199657	0.482498	8.70E-14
rs12446515	T	C	-0.0334207	0.00213589	0.322889	3.50E-55
rs34682685	A	G	0.0337428	0.00326987	0.104198	5.80E-25
rs4471666	G	T	-0.022461	0.00396699	0.069488	1.50E-08
rs28577186	A	G	-0.0163428	0.00211672	0.664571	1.20E-14
rs2925979	C	T	-0.0322092	0.00217095	0.699976	8.50E-50
rs742036	A	G	-0.0143632	0.00205112	0.375259	2.50E-12
rs112381903	T	A	0.0231649	0.00400864	0.06731	7.50E-09
rs117431393	G	A	0.0312254	0.00537977	0.035446	6.50E-09
rs12185242	C	A	0.0175265	0.00199898	0.454836	1.80E-18
rs1801689	C	A	-0.06613	0.00577722	0.030543	2.40E-30
rs60856912	T	G	0.0248329	0.00270829	0.162906	4.80E-20
rs9889402	A	G	0.0122827	0.00222926	0.728192	3.60E-08
rs4969179	G	T	-0.0177529	0.00203649	0.60445	2.80E-18
rs12948505	T	C	0.0138887	0.00251214	0.194119	3.20E-08
rs2304969	T	G	-0.0161469	0.0028501	0.144932	1.50E-08
rs1292065	G	C	-0.0139237	0.00218669	0.709244	1.90E-10
rs11078597	C	T	0.0191708	0.0025513	0.186613	5.70E-14
rs9902027	T	C	-0.0151609	0.00238645	0.77437	2.10E-10
rs7215055	G	A	0.0389233	0.00410062	0.062892	2.30E-21
rs10775406	G	A	0.0206571	0.00232578	0.759694	6.60E-19
rs145947882	C	A	0.137333	0.0063668	0.025952	3.40E-103
rs11664106	T	A	-0.0125831	0.00210816	0.374102	2.40E-09
rs867939	A	G	-0.0136048	0.00202516	0.57621	1.80E-11
rs7239575	C	T	-0.0160767	0.00199133	0.490494	6.80E-16
rs2187114	A	G	-0.0185151	0.00330375	0.101024	2.10E-08
rs6506033	T	C	-0.0228948	0.00383903	0.072523	2.50E-09
rs921971	C	T	0.01565	0.00225602	0.265901	4.00E-12
rs62102718	T	A	0.0202348	0.00220142	0.286196	3.90E-20
rs5112	G	C	0.0684501	0.00213412	0.533428	1.00E-200
rs62112763	G	C	0.01935	0.00200786	0.438929	5.60E-22

rs483808	T	C	-0.0136636	0.00218517	0.701973	4.00E-10
rs2305746	G	A	0.0282544	0.0039744	0.933319	1.20E-12
rs8102873	T	C	0.0123302	0.00201474	0.584919	9.40E-10
rs10405944	C	T	-0.013209	0.00201995	0.483077	6.20E-11
rs483082	T	G	0.0862119	0.00234474	0.23535	1.00E-200
rs116843064	A	G	-0.226505	0.00721009	0.019327	1.00E-200
rs58542926	T	C	-0.103184	0.00378074	0.074608	5.30E-164
rs188247550	T	C	-0.134286	0.00913128	0.013117	5.90E-49
rs62128802	T	C	-0.0158746	0.00257782	0.183216	7.40E-10
rs2081194	C	G	-0.0214115	0.00207674	0.600934	6.30E-25
rs2092203	T	C	0.0136756	0.00199323	0.481154	6.80E-12
rs6073958	C	T	0.0556952	0.00249344	0.198674	1.60E-110
rs8126001	T	C	-0.0163778	0.00199681	0.489576	2.40E-16
rs149142833	T	C	0.0169358	0.00276509	0.156359	9.10E-10
rs6028716	A	G	-0.0128217	0.00228302	0.258428	2.00E-08
rs55966194	G	C	-0.0178509	0.00221245	0.281997	7.10E-16
rs151235402	T	C	0.0521833	0.00815144	0.015827	1.50E-10
rs7274718	A	G	0.0159491	0.00202919	0.598516	3.80E-15
rs394872	T	C	0.0111807	0.00200285	0.536237	2.40E-08
rs6517522	C	T	-0.0129031	0.00199242	0.498138	9.40E-11
rs140288	A	G	-0.0132623	0.00200841	0.566711	4.00E-11
rs134551	T	C	-0.0116536	0.00211111	0.335438	3.40E-08
rs2267373	T	C	0.0215638	0.00202145	0.580693	1.40E-26
rs5755799	G	C	0.0118859	0.00200343	0.453747	3.00E-09
rs2071887	A	T	0.016312	0.00209671	0.344804	7.30E-15
rs4253750	C	T	0.0177576	0.00243701	0.214455	3.20E-13

A1: Effect Allele; A2: Alternative Allele; EAF: Effect Allele Frequency; Beta: beta coefficient; SE: Standard error of beta coefficient; *p*: *p*-value of the meta-analysis

Table S4. SNPs of total cholesterol extracted from GLGC with statistically significant threshold

rsID	A1	A2	BETA	SE	EAF	*p* value
rs11802413	T	C	0.0287	0.0035	0.5369	1.58E-14
rs11591147	T	G	-0.3341	0.0173	0.01715	8.83E-86
rs646776	T	C	0.1272	0.0042	0.7876	4.78E-187
rs558971	G	A	0.0398	0.0036	0.5303	7.02E-28
rs2642438	G	A	0.037	0.004	0.7454	1.28E-18
rs7534572	G	C	0.0629	0.0055	0.69	3.60E-28
rs6603981	T	C	0.0351	0.0043	0.8061	7.85E-15
rs7551981	T	G	0.0358	0.0037	0.595	7.50E-22
rs4988235	A	G	-0.0308	0.004	0.5237	3.97E-14
rs11694172	G	A	0.0277	0.0041	0.2164	1.95E-09

rs2030746	T	C	0.0199	0.0037	0.3984	3.60E-08
rs17526895	G	A	-0.042	0.0067	0.07784	5.78E-09
rs2287623	A	G	-0.0273	0.0036	0.595	4.09E-12
rs9306897	C	T	-0.0488	0.0037	0.6966	7.51E-37
rs780093	C	T	-0.0515	0.0036	0.5871	2.59E-42
rs6544713	C	T	-0.0773	0.004	0.7058	1.69E-81
rs6709904	G	A	-0.0545	0.0083	0.1135	8.39E-10
rs11563251	T	C	0.0368	0.0059	0.1253	1.27E-09
rs515135	C	T	0.1238	0.0046	0.7823	6.38E-151
rs13315871	A	G	-0.0355	0.0061	0.08047	3.48E-08
rs7616006	G	A	-0.0315	0.0036	0.4446	8.41E-17
rs7640978	T	C	-0.0376	0.0066	0.1055	1.66E-08
rs6818397	G	T	-0.0254	0.0039	0.5871	9.51E-11
rs4530754	A	G	0.0228	0.0035	0.5818	1.68E-09
rs12916	C	T	0.0684	0.0036	0.4314	4.55E-74
rs6882076	C	T	0.0508	0.0037	0.6662	5.35E-41
rs2814982	T	C	-0.0441	0.0057	0.1069	3.68E-15
rs3757354	T	C	-0.0348	0.0042	0.2098	2.22E-15
rs9272775	C	T	0.0317	0.0055	0.2823	2.13E-08
rs11153594	T	C	-0.029	0.0036	0.3918	1.27E-14
rs2315065	A	C	0.1102	0.0158	0.08707	1.10E-11
rs1800562	A	G	-0.0565	0.0077	0.04617	1.91E-12
rs9391858	G	A	0.0495	0.005	0.1939	7.20E-22
rs9376090	C	T	-0.0254	0.004	0.2718	2.60E-09
rs112201728	T	C	0.0581	0.0099	0.05805	1.20E-08
rs11753995	A	G	0.0489	0.0048	0.1464	1.84E-23
rs1997243	G	A	0.0332	0.005	0.1306	2.72E-10
rs12670798	C	T	0.0364	0.0041	0.2243	9.48E-17
rs2073547	G	A	0.0456	0.0047	0.1939	3.83E-21
rs2737252	A	G	-0.0331	0.0039	0.2559	1.63E-16
rs9987289	G	A	0.0842	0.0063	0.9248	1.84E-36
rs2954029	T	A	-0.0622	0.0035	0.4683	2.42E-65
rs7832643	T	G	0.0289	0.0037	0.405	3.12E-13
rs10088180	G	A	-0.0228	0.004	0.6781	6.02E-10
rs4738684	G	A	-0.0392	0.0037	0.6478	1.12E-23
rs1883025	T	C	-0.0671	0.0042	0.2427	5.75E-53
rs581080	C	G	0.0377	0.0047	0.8206	1.02E-13
rs11789603	T	C	0.0427	0.0062	0.08971	1.44E-11
rs2066714	C	T	0.0442	0.0076	0.1201	1.14E-08
rs579459	C	T	0.062	0.0044	0.215	8.83E-42
rs3780181	G	A	-0.0442	0.0071	0.05277	6.67E-10
rs12412743	T	C	-0.0298	0.0047	0.153	6.98E-10

rs10904908	G	A	0.025	0.0036	0.4538	2.60E-11
rs2255141	G	A	-0.0314	0.0039	0.6807	6.51E-16
rs10900221	A	G	0.0255	0.0041	0.2731	7.96E-09
rs1535	G	A	-0.0497	0.0037	0.3628	8.62E-39
rs4752805	G	A	0.0251	0.0041	0.2467	1.62E-09
rs10832962	T	C	0.0315	0.0039	0.719	1.54E-14
rs964184	C	G	-0.1214	0.0076	0.719	2.84E-55
rs11220462	A	G	0.0474	0.0058	0.1425	5.49E-15
rs3184504	C	T	0.0318	0.0037	0.5343	1.62E-17
rs10773003	A	G	0.0369	0.0058	0.08839	4.08E-09
rs2244608	G	A	0.0313	0.0037	0.3391	9.62E-18
rs6573778	C	T	-0.0263	0.0039	0.529	2.96E-11
rs10468017	T	C	0.0617	0.004	0.2757	7.23E-48
rs633695	G	A	0.0433	0.0058	0.285	1.05E-14
rs2000999	A	G	0.0617	0.0044	0.1847	6.80E-41
rs247616	T	C	0.0499	0.004	0.2929	4.47E-32
rs6504872	T	C	0.025	0.0035	0.4723	6.99E-12
rs2886232	C	T	-0.0358	0.0062	0.8799	3.87E-08
rs314253	C	T	-0.0233	0.0037	0.3351	2.81E-10
rs2156552	T	A	0.057	0.0047	0.8219	1.25E-31
rs2228603	T	C	-0.1217	0.0069	0.07124	1.05E-62
rs281393	T	C	-0.0322	0.0055	0.3734	4.26E-08
rs8103315	A	C	0.0422	0.0055	0.1359	5.94E-15
rs75687619	T	G	0.1592	0.0153	0.02375	3.61E-22
rs6511720	T	G	-0.1851	0.0059	0.09763	1.00E-200
rs7412	T	C	-0.3736	0.0096	0.06596	1.00E-200
rs6016373	G	A	-0.0319	0.0036	0.3734	1.00E-17
rs1800961	T	C	-0.1062	0.0101	0.0343	1.34E-24
rs2277862	T	C	-0.0349	0.0052	0.1319	5.26E-11
rs2235367	G	A	0.0357	0.0035	0.4565	7.22E-25
rs181360	G	T	-0.0278	0.0043	0.1992	7.32E-10
rs138777	G	A	-0.0214	0.0037	0.6517	4.74E-08
rs4253772	T	C	0.0322	0.0058	0.1187	9.85E-09

A1: Effect Allele; A2: Alternative Allele; EAF: Effect Allele Frequency; Beta: beta coefficient; SE: Standard error of beta coefficient; *p*: *p*-value of the meta-analysis

Table S5. Causal estimates of genetically predicted plasma lipid levels on the risk of AA, stratified by gender

Exposure	SNP (n)	Method	OR (95% CI)	*p* value
HDL-C (Female)	154	Inverse variance weighted	1.90 (1.31-2.76)	0.001
		Weighted median	1.63 (0.94-2.83)	0.081
		Weighted mode	1.23 (0.72-2.11)	0.450
		MR Egger	1.71 (0.90-3.28)	0.105
HDL-C (Male)	110	Inverse variance weighted	2.27 (1.47-3.51)	0.0002
		Weighted median	1.98 (1.02-3.85)	0.043
		Weighted mode	1.75 (0.92-3.30)	0.090
		MR Egger	1.49 (0.71-3.11)	0.291
LDL-C (Female)	97	Inverse variance weighted	0.66 (0.53-0.81)	7.55E-05
		Weighted median	0.69 (0.53-0.90)	6.00E-03
		Weighted mode	0.71 (0.56-0.91)	7.09E-03
		MR Egger	0.67 (0.50-0.91)	1.28E-02
HDL-C (Male)	71	Inverse variance weighted	0.67 (0.52-0.85)	0.001
		Weighted median	0.66 (0.49-0.88)	0.004
		Weighted mode	0.74 (0.56-0.97)	0.030
		MR Egger	0.68 (0.48-0.96)	0.033
TG (Female)	116	Inverse variance weighted	0.80 (0.67-0.96)	0.014
		Weighted median	0.82 (0.63-1.08)	0.164
		Weighted mode	0.84 (0.65-1.10)	0.208
		MR Egger	0.85 (0.64-1.13)	0.273
TG (Male)	80	Inverse variance weighted	0.81 (0.71-0.92)	0.002
		Weighted median	0.80 (0.65-0.98)	0.028
		Weighted mode	0.82 (0.68-1.00)	0.052
		MR Egger	0.88 (0.72-1.08)	0.217

LDL-C, low-density lipoprotein cholesterol; TC, total cholesterol; TG, triglyceride; HDL-C, high-density lipoprotein cholesterol; AA, Aortic aneurysm; CI, confidence interval; OR, odds ratio.

Table S6. Causal estimates of genetically predicted plasma lipid levels on the risk of AD, stratified by gender

Exposure	SNP (n)	Method	OR (95% CI)	*p* value
HDL-C (Female)	154	Inverse variance weighted	1.11 (0.52-2.37)	0.787
		Weighted median	2.12 (0.57-7.95)	0.264
		Weighted mode	1.54 (0.38-6.34)	0.548
		MR Egger	1.61 (0.43-5.99)	0.479
HDL-C (Male)	110	Inverse variance weighted	1.06 (0.38-2.97)	0.908
		Weighted median	2.00 (0.41-9.80)	0.391
		Weighted mode	0.93 (0.15-5.65)	0.935
		MR Egger	0.93 (0.16-5.39)	0.939
LDL-C (Female)	97	Inverse variance weighted	0.84 (0.56-1.24)	0.379
		Weighted median	0.63 (0.33-1.20)	0.158
		Weighted mode	0.63 (0.33-1.22)	0.176
		MR Egger	0.83 (0.46-1.48)	0.525
HDL-C (Male)	71	Inverse variance weighted	0.97 (0.60-1.57)	0.912
		Weighted median	0.72 (0.35-1.46)	0.360
		Weighted mode	0.71 (0.37-1.36)	0.305
		MR Egger	0.85 (0.43-1.68)	0.640
TG (Female)	116	Inverse variance weighted	0.92 (0.62-1.37)	0.679
		Weighted median	1.20 (0.63-2.29)	0.578
		Weighted mode	1.05 (0.53-2.11)	0.884
		MR Egger	1.07 (0.56-2.02)	0.842
TG (Male)	80	Inverse variance weighted	0.96 (0.69-1.33)	0.808
		Weighted median	1.13 (0.72-1.80)	0.590
		Weighted mode	1.13 (0.73-1.76)	0.587
		MR Egger	1.22 (0.74-2.00)	0.436

LDL-C, low-density lipoprotein cholesterol; TC, total cholesterol; TG, triglyceride; HDL-C, high-density lipoprotein cholesterol; AD, Aortic dissection; CI, confidence interval; OR, odds ratio.
